# Two Mechanisms: The Role of Social Capital and Industrial Pollution Exposure in Explaining Racial Disparities in Self-Rated Health

**DOI:** 10.3390/ijerph13101025

**Published:** 2016-10-19

**Authors:** Kerry Ard, Cynthia Colen, Marisol Becerra, Thelma Velez

**Affiliations:** 1 School of Environment and Natural Resources, Ohio State University, Columbus, OH 43210, USA; becerra.21@osu.edu (M.B.); velez.71@osu.edu (T.V.); 2 Department of Sociology, Ohio State University, Columbus, OH 43210, USA; colen.3@osu.edu

**Keywords:** neighborhood effects, racial disparities, health disparities, social capital, industrial air pollution

## Abstract

This study provides an empirical test of two mechanisms (social capital and exposure to air pollution) that are theorized to mediate the effect of neighborhood on health and contribute to racial disparities in health outcomes. To this end, we utilize the Social Capital Benchmark Study, a national survey of individuals nested within communities in the United States, to estimate how multiple dimensions of social capital and exposure to air pollution, explain racial disparities in self-rated health. Our main findings show that when controlling for individual-confounders, and nesting within communities, our indicator of cognitive bridging, generalized trust, decreases the gap in self-rated health between African Americans and Whites by 84%, and the gap between Hispanics and Whites by 54%. Our other indicator of cognitive social capital, cognitive linking as represented by engagement in politics, decreases the gap in health between Hispanics and Whites by 32%, but has little impact on African Americans. We also assessed whether the gap in health was explained by respondents’ estimated exposure to toxicity-weighted air pollutants from large industrial facilities over the previous year. Our results show that accounting for exposure to these toxins has no effect on the racial gap in self-rated health in these data. This paper contributes to the neighborhood effects literature by examining the impact that estimated annual industrial air pollution, and multiple measures of social capital, have on explaining the racial gap in health in a sample of individuals nested within communities across the United States.

## 1. Introduction

There is a robust literature evidencing health disparities in the United States between non-Hispanic Whites (herein referred to as Whites) and racial and ethnic minorities [1,2,3]. With results showing that compared to Whites, African Americans have greater rates of cardiovascular disease [4], mortality [5], stress [3], low-birthweight [6], and life expectancy [7]. Although Hispanics, on average, experience lower overall mortality rates compared to Whites [8] they have been shown to have a higher prevalence of chronic diseases and risk-factors such as: diabetes [9], liver disease [10], HIV/AIDS [10], obesity [11] and poor self-rated health [12]. Traditionally, scholars interested in unraveling these disparities have focused on personal characteristics [13]. However, even when controlling for individual-level explanatory variables, such as age, sex, health behaviors, and access to care, racial gaps in health remain [14,15,16]. These findings have prompted researchers to consider the independent effects of place on health, with results suggesting that controlling for residential context can reduce racial gaps in self-rated health by 15%–76% [13]. 

Within the past two decades the “neighborhood effects” literature has grown to provide substantial evidence that the area in which people reside has an impact on their health, independent of individual-level factors [13,15]. Nevertheless, social problems are often bundled together at the neighborhood-level and hard to untangle [16], leaving today’s researchers still struggling to understand what characteristics of “place” are most influential in explaining health outcomes. This question takes on particular salience in the health disparities literature. The persistence of racial segregation in America’s communities means that racial groups are exposed to different social and physical environments. A good deal of research suggests that predominately non-White communities pose a greater health risk to their residents. For example, studies show that in more racially segregated metro areas, African Americans are exposed to greater health risk from air toxins compared to Whites [17] and from 1994 to 2004 an average African American in the United States was exposed to roughly twice the health risk from industrial air pollution than an average White person, with middle-class African Americans more exposed than lower-class Whites [18]. Furthermore, while Hispanics have on average less exposure to industrial air pollutants at a national-level, as they tend to live in areas with lower than the national average, they do face higher exposure than Whites within the cities in which they live [19]. Studies examining the health effects of neighborhood-level pollution have shown being in these areas is associated with worse cognitive outcomes [20], greater rates of diabetes [21], greater rate of hospital admissions [22], and higher mortality and morbidity generally [23]. However, only a small number of these studies have examined the role these exposures have on the racial-gap in health, with very few to none controlling for individual-level covariates in a nationally representative sample in the U.S. [24].

In addition to greater exposure to physical hazards, like air pollution, communities that are predominately non-White are argued to be more exposed to greater social hazards that impact health, such as crime [25] and joblessness [26]. These cumulative social insults on health have often been examined in the neighborhood effects literature using the Social Organization Theory. This theory, first developed in 1942 by early Chicago School sociologists Shaw and McKay, holds that neighborhood disadvantage leads to difficulties in establishing and maintaining order, inhibiting residents’ ability to organize for collective benefit, and making them more vulnerable to risk [27]. In the 1990s the concept of social capital gave scholars a useful vehicle through which to unpack what particular aspects of social (dis)order are most influential on health and the research showing associations between different types of social capital and a variety of health outcomes grew quickly [28]. Despite the growing association between health and social capital, and evidence that the amount, and type, of social capital varies by the racial characteristics of communities, few have examined the extent to which social capital mediates racial disparities in health [29]. As Gilbert and Dean [30] note, “… when examining social capital and health race/ethnicity has been left out of the conversation”. 

It is clear that racial differences in health cannot be merely explained by individual-level variables. However, because individuals are spatially separated based on their race and ethnicity in the U.S. individuals within the same racial/ethnic group are exposed to similar environmental and social health risks. In order to understand which part of an individual’s neighborhood is most meaningful for health we need to examine the independent role of multiple mechanisms on the same population while simultaneously considering individual covariates and community context. The analyses presented below will take a step toward that goal by using a national survey of individuals nested within communities to examine to what extent the racial and ethnic gap in self-rated health can be explained by different dimensions of social capital and pollution exposure within communities while controlling for individual-level confounders. 

### 1.1. Social Capital and Health Inequality

Public health scholars have readily adopted the concept of social capital from sociology, showing it reduces the odds of all-cause mortality [31], chronic disease [32], and a multitude of other health outcomes [33]. In a recent meta-analysis examining studies of social capital and health, Gilbert et al. [33] notes that social capital has been shown to increase the odds of good health by 27%, with some aspects of social capital, such as trust and reciprocity, increasing the odds of good health by 32% and 39%, respectively [33]. However, as this scholar and others note, there has been less attention given to the role social capital plays in explaining racial disparities in health. Conceptually, if levels, and types, of social capital vary by neighborhood, with some neighborhoods characterized by a predominance of a specific racial group, then we would expect that once we controlled for social capital some of the racial disparity in health outcomes may be diminished [29].

There has been a variety of organizing frameworks utilized by scholars to operationalize the concept of social capital. The most commonly used was posed by sociologist Robert Putnam who suggested that social ties between individuals of similar social standing (Bonding Social Capital) should be examined separately from social ties between groups of individuals with different social standings (Bridging Social Capital) [34]. The health researchers that utilize this framework argue that bonding social capital provides emotional and material support that could potentially help individuals buffer the precursors of negative health outcomes. Bridging measures, on the other hand, can help provide individuals with more diverse health information and useful social connections [30]. Some social scientists have argued for a sub-classification of bridging capital called “Linking Social Capital”, which specifies social ties that link individuals, or groups, of varying social power. This type of social capital is argued to create trust in formal institutions and feelings of empowerment which translate to less stress and better health [35,36,37,38]. A handful of studies have examined how these types of social capital have varied across racial groups. Using General Social Survey data from 2002, Liu et al. [39] found that African Americans were more likely to have bonding social capital, as measured by church attendance, than Whites. While Whites were more likely to have bridging or linking social capital, as measured by participation in collective activities [39]. All of these studies follow from sociologist Carol Stack’s 1974 classic argument that because African Americans, and by extension other non-Whites, are segregated into more disadvantaged communities they require strong “bonding” capital, in today’s vernacular, to compensate for the lack of structural/institutional support [40]. 

Several health scholars have reasoned these frames, which stem largely from the field of sociology, are inadequate to understand the role of social capital in health. Rather, they argue, a distinction needs to be made between Cognitive and Structural Social Capital [38,41,42]. Cognitive Social Capital has been defined as what people think and feel and is reflected in measures of perceptions and values [42,43]. For example, greater feelings of trust in one’s community have been shown to be related to better self-rated health [44]. Perceptions of shared trust and values are thought to benefit individuals’ health by increasing feelings of security and esteem [45]. Structural Social Capital is defined as what people do, which can be objectively measured as an individual’s activity in organizations and networks [42]. This type of social capital is thought to benefit health by providing individuals with greater access to institutions and novel health-related knowledge [46]. Hovick et al. [47] found support for this perspective by demonstrating greater activity in organizations was significantly related to seeking health information [47]. Level of Cognitive and Structural Social Capital are expected to vary by race due to the pervasive racial discrimination experienced by non-White individuals in American society. Perceptions of racial discrimination have been linked to lower levels of trust [48]; increased depression, anxiety, and lack of security which create a withdraw from social activities [49], all of which would suggest non-Whites would experience lower overall Cognitive and Structural Social Capital. In this paper, we employ a scheme previously developed by Uphoff [42] which overlaps the traditional sociological framework that distinguishes between Bonding, Bridging and Linking Social Capital with the public health framework that denotes Cognitive and Structural Social Capital. This schema provides us with five components of social capital covering different levels of bonding, bridging, and linking social capital and two modes of measurement: cognitive and structural. 

### 1.2. Pollution and Health Inequality

There is a great deal of research evidencing the fact that non-White Americans are at a higher risk from exposure to environmental pollution. For example, a survey of 800 residents in Miami-Dade County found that African Americans had higher levels of the pesticides DDT and DEE even when compared to Whites in the same social class [50]. In the Southern California Basin, Brajer and Hall [51] found that Hispanics, African Americans, and those in the lowest economic stratum had the highest rates of exposure to fine particulate matter and sulfur dioxide. A more recent study in El Paso, Texas shows that census block groups with the highest proportion of Hispanics were 3.6 times more likely to be exposed to toxic air pollutants than those areas with the fewest Hispanics [52]. These area level studies strongly support the theory that non-White populations experience a greater health risk from environmental pollutants. Similar results have been found for the few individual-level studies examining how risk for environmental exposure contributes to racial disparities in health. For example, researchers found that in a sample of households from Miami, Florida, Hispanic and African American individuals had a greater risk of cancer from Hazardous Air Pollutants (HAP), even when controlling for household socioeconomic (SES) factors [24]. When this study was repeated in a sample of households in the Greater Houston metro area in Texas, researchers showed the same findings: African American and Hispanic individuals had a higher cancer risk from HAPs even when controlling for household SES. Greater exposure to such toxins has been shown to be related to worse self-rated health [53,54,55,56]. For example, a study on the perceived health effects from daily air pollution changes in Montreal, Quebec found a significant drop in self-rated health with increases in outdoor PM_2.5_ [53]. Another study exploring air pollution exposure and self-reported cardiovascular disease yielded a positive correlation between increases in fine particulate matter and adverse cardiovascular outcomes [54]. Similarly, a study focusing on air pollution effects in the elderly population of China found a 17% increase in self-rated poor health with a one-level rise in air pollution [56].

While both individual and community-level studies in the U.S. show that racial and ethnic minorities are at a greater health risk from air pollution, and exposure to air pollution is related to worse self-rated health, how community-level exposure relates to health outcomes has rarely been studied at the individual-level. Chakraborty et al. [24] has noted that most studies examining the role of air pollution in explaining racial health disparities have been at the community-level and thus unable to control for individual demographics related to mortality and morbidity, like age, sex, education and income. Those few studies that have been done have shown an interesting relationships between race, pollution and health. For example, Grineski [57] examined the effects of particulate matter (PM_2.5_) and Nitrogen Dioxide on hospital admissions for asthma, Chronic Obstructive Pulmonary Disease, and Congestive Heart Failure in El Paso County, Texas. The authors found that Hispanics were at a lower risk for hospital admissions for all three diseases compared to Whites at the same level of exposures to Nitrogen Dioxide. However, they also found Hispanics were more likely than Whites to be admitted for Congestive Heart Failure when PM_2.5_ levels were high. Another study done in Atlanta, Georgia showed the associations between ozone and preterm birth were significantly stronger for children born to African American mothers [58]. On the other hand, a study done in Arkansas showed that the effect of PM_2.5_ on cardiovascular and respiratory emergencies was stronger for Whites. If the relationship between race and health is mediated by pollution exposure, why would we see that these relationships vary by race? One possibility is that there are other social factors unaccounted for, such as social capital. This study works to elucidate the independent role that estimated exposure to air toxins and social capital has on explaining the racial gap in self-rated health in the same population. This work will help isolate the mediating effects of each of these mechanisms so that future researchers can explore how their interaction of them explains health outcomes. 

## 2. Materials and Methods

### 2.1. Description of Data and Measures

For the current study, we combined data from two existing secondary sources. Information regarding social capital, as well as health, was obtained from the Robert Putnam’s 2000 Social Capital Benchmark Survey (SCBS), while estimates of industrial pollutant toxicity originated from the United States Environmental Protection Agency’s (EPA) Risk-Screening Environmental Indicator Geographically Modeled Data (RSEI-GM). The SCBS is a national, cross-sectional study that was originally designed to explore sources of, and outcomes associated with, a variety of social capital constructs. Data were collected from telephone surveys from July to November 2000, with the exception of West Oakland, California, which was surveyed from December 2000 to February 2001. The majority (90%) of participants resided within one of 42 communities across 29 states, while a small proportion (10%) formed the basis of a national sample. Local organization were sought out to define what specific areas defined their community boundaries. These boundaries tended to be defined by a county or contiguous county, however in some cases the entire state or sections of the state, such as South Dakota and Montana. There was an average of 648 people in each community, with a minimum of 357 and a maximum of 2825. These are equivalent in size to most census block groups, which average between 600 and 3000 people. For a more detailed description of the methodology and break down of communities, please see the Social Capital Community Benchmark Survey Executive Summary [59]. Because the geographic boundaries of a neighborhood/community were defined by these local organizations they did not necessarily fall within census boundaries, however in order to match community characteristics to estimates of individual health and exposure to pollution we needed more precise estimates of where an individual lived in these communities. To achieve this we utilized the restricted version of the SCBS data, which provides the census block group a respondent lived in during the year 2000. SCBS individuals in our sample lived in 13,809 census block groups; the average number of survey respondents in a census block group was two, with a minimum of one and a maximum of 460 sample. 

The RSEI-GM data (version 2.1.5) are derived from 17,603 industrial facilities that are required to submit information to the EPA on the amount of 589 toxic air pollutants emitted over the past year [60]. Estimates about the type and toxicity of pollutants within a 101 km radius around each existing facility was determined using plume modeling which considers the height of the facility’s smoke stake, the velocity with which pollutants were emitted, typical wind patterns, and a number of other salient factors [60]. These estimates were overlaid with a grid of the continental U.S. that was broken down into over 14 million one-by-one kilometer grid cells, with each grid cell being assigned an estimate of the total amount of an air pollutant (μm^3^) expected to affect that area over the past year. Each one of these toxins was then weighted by its toxicity to human health. This toxicity was assigned by the EPA using epidemiological information with the intention to make toxicity risk commensurate across disease category [60]. This detailed process resulted in the creation of a toxicity-weighted pollution exposure estimate for every kilometer square in the United States. Following Ard [20] the estimates for these one-by-one kilometer grid cells were overlaid with census boundaries and weighted by the amount of area they contributed to census block groups and then aggregated up to the block group level. This process creates an estimate of the toxicity-weighted air pollution expected to be in that block group in the past year. This allows the toxicity of one block group to be compared to the toxicity of another block group. In concordance with prior research efforts using these data we employed a version of this indicator of industrial pollutant toxicity that is divided into deciles [20].

The dependent variable of interest for the current study is self-rated health. Participants in the SCBS were asked, “Compared to other people your age, how would you describe your health?” and were able to select one of five response categories (excellent, very good, good, fair, or poor). This type of global health measure possesses a number of methodological strengths. First, it is widely used so it allows us to compare our results to a large proportion of existing studies concerning the role social capital is likely to play in the production of health as well as empirical work on the causes and consequences of racial/ethnic disparities in health. Second, self-rated health has been shown to be highly predictive of subsequent morbidity and mortality, more so than direct examination by a physician [61,62]. Finally, such an indicator is easily understood, interpreted, and answerable by a wide variety of respondents, even those with low levels of educational attainment or those with limited language skills [63,64]. In the following analysis, we use self-rated health as an ordered variable but results were robust to this variable dichotomized.

We relied on seven different measures of social capital to capture the different dimensions of such a complex social phenomenon. These are described, in-depth, in Table 1. These indicators were originally developed by Putnam and colleagues [58] based on existing theoretical knowledge of distinctions among various types of social capital and refined using a multi-step factor analytic process. More complete details regarding the methodology used to construct these social capital measures can be found in the Social Capital Benchmark Study methodology documents [58]. In order to combine both the public health and sociological social capital frameworks, we categorized social capital measures into the following five components: bonding, bridging, linking, cognitive, and structural, which are detailed in Table 1. These components were previously used in a systematic review of the literature examining the relationship between socioeconomic inequality and health [26].

### 2.2. Analytic Strategy

We rely on multilevel regression analysis to estimate the extent to which differential exposure to industrial pollutants and social capital contribute to existing racial/ethnic disparities in health [59,60]. Although the Intraclass Correlation Coefficient is relatively small (ranging from 0.003 to 0.004 in our models), we still prefer to use multilevel models as the clustering of respondents in communities violates the assumption of independent error required for simpler models. In order to account for clustering within the data (individuals within communities) as well as the nature of the response categories for the dependent variable of interest, a series of ordered logit models with random intercepts predicting self-rated health were estimated, with success probabilities determined by the logistic cumulative distribution function. The composite statistical model can be written as follows:

(1) $$logit[\mathrm{Pr}(y_{ij}\leq s|x_{ij},\varsigma_{j})]=\left(\beta_{1}+\varsigma_{j}\right)+\beta_{2}x_{2ij}+\ldots \;+\beta_{n}x_{nij}+\epsilon_{ij}$$

where *y_ij_* indicates the health status of individual *i* within community *j*, *β*_1_ represents the average intercept, *ς_j_* indicates the individual-specific error component that remains constant across communities, ϵ*_ij_* stands for the community-specific error component, *β*_2_ − *β_n_* are regression coefficients for individual *i* within community *j*, and *x*_2_ − *x_n_* are the values of each predictor for individual *i* within community *j*. The random intercepts are assumed to be independent and normally distributed across individuals and independent of the covariates x*_ij_*. For area-specific variables, the value of *x_n_* will be constant across all individuals in community *j*.

First, we individually examined how each dimension of social capital contributed to racial/ethnic disparities in self-rated health. Once particularly salient social capital predictors were identified, we incorporated them into a set of final regression models that also included our measure of industrial pollutant toxicity. Multilevel regression analysis was employed to account for clustering within our data (individuals within communities), thus violating the independence of error assumption. 

For all models, a number of sociodemographic control variables were included to, at least in part, adjust for potential confounding. These include age, sex (male vs. female), region of residence (Northeast, Midwest, South, or West), marital status (never married, currently married, widowed, or divorced/separated), and educational attainment (some high school, high school graduate, some college, associate’s degree, college graduate, or graduate school). Robust standard errors were calculated using the Huber/White correction method and clustered at the community level. Complex sampling weights that account for differential selection into the SCBS as well as the oversampling of racial/ethnic minorities were consistently applied.

Of the original 29,733 SCBS participants/respondents, 1951 of these had moved into their community within the year and thus it could not be assumed they had been exposed to the estimated annual pollution. However, after running sensitivity analyses, we found no appreciable change in our findings if we dropped them, so opted to leave them in. An additional 513 respondents had incomplete block group information, preventing us from linking geocoded pollution data from the RSEI-GM to sociodemographic and health data from the SCBS; 882 respondents were also excluded due to missing data on either the dependent variable (self-rated health) or any of the independent variables of interest (social capital). This resulted in a final analytic sample of 26,387 individuals nested within 42 geographic areas. 

## 3. Results

Seventy-seven percent of SCBS respondents identify as White and 14% African American. Nine percent are categorized as Hispanic (Table 2); this group includes those of Mexican, Puerto Rican, Cuban and “other” nationality. While preferable to analyze these groups separately, we were unable to do so because of inadequate sample size for these subgroups. Future work should consider how social capital varies within racial groups. Descriptive statistics reveal clear racial/ethnic disparities in health. While 63% of Whites rate their health as being excellent or very good, only 52% of African Americans and 46% of Hispanics do so. Table 2 also shows that African Americans had the greatest amount of exposure to toxic pollution, with an average decile of exposure of 6.53; Hispanics had the lowest average exposure decile, 4.77, and Whites were in the middle with an average exposure of 5.48. Subsequent bivariate regression analyses (results not shown) revealed that the racial/ethnic disparities in pollution exposure displayed in Table 2 were statistically significant for African Americans (*t* = 3.32, *p* < 0.002) and marginally significant for Hispanics (*t* = −1.85, *p* < 0.071). Compared to Whites, Hispanics consistently rank lower on all social capital indicators. African Americans, on the other hand, display lower levels of social capital than Whites on some indicators (informal social participation, general social trust, and electoral politics) but higher levels on others (faith-based social capital, organized social participation, political activism, and formal group involvement). It is important to note that the first four social capital variables: informal social participation, faith-based social capital, social trust, and organized social participation, have been standardized using values from the national subsample of the SCBS as a referent group. Therefore, negative values indicate levels of social capital that fall below the U.S. mean, while positive values represent levels of social capital that are higher than national averages. 

There are also clear racial/ethnic differences on a number of important social and demographic variables that we treat as potential confounders in subsequent multilevel models (Table 2). The largest proportion of African Americans (31%) reside in the South, while almost half of Hispanics (46%) live in the West. These racial/ethnic disparities in region of residence are reflective of long-standing national trends [65,66]. Only 39% of African Americans and 53% of Hispanics are currently married compared to 61% of their White counterparts. Racial/ethnic disparities in educational attainment are quite apparent, particularly among Hispanic respondents. Forty-two percent of Hispanics report never having finished high school. This is true of only 9% of Whites and 14% of African Americans. At the opposite end of the educational spectrum, more than 28% of Whites have at least a college degree, compared to only 16% of African Americans and 10% of Hispanics.

Results from multilevel ordered logistic regression models illustrating how different dimensions of social capital explain racial/ethnic disparities in self-rated health are presented in Table 3. Model 1 reveals that African Americans and Hispanics were 28% [1 − exp(−0.333)] and 38% [1 − exp(−0.475)], respectively, less likely to be in good health than Whites. It should be noted that these racial/ethnic disparities are highly statistically significant even after controlling for all additional covariates (age, sex, region of residence, marital status, and educational attainment) as well as after controlling for education, income, and social capital measure at the community-level (results not shown). In Models 2 through 8, we individually entered each social capital indicator to the initial model (Model 1) to estimate the extent to differential access to different dimensions of social capital could account for these racial disparities in self-rated health. Although all social capital indicators were significantly associated with self-rated health, only two—general social trust (Model 4) and participation in electoral politics (Model 8)—accounted for substantial amount of racial/ethnic disparities in self-rated health. Both of these measures are classified as cognitive social capital indices, one bridging and one linking, respectively.

Comparing results/findings from Model 2 to Model 1, it becomes evident that industrial pollutant toxicity does not account for the stark racial/ethnic disparities in self-rated health that exist in our data. Moreover, the magnitude of the regression coefficient for air pollution is quite small and not statistically significantly different from zero (*p* < 0.05). Results concerning our two social capital measures (Models 3 and 4) are decidedly different.

**Figure 1:**
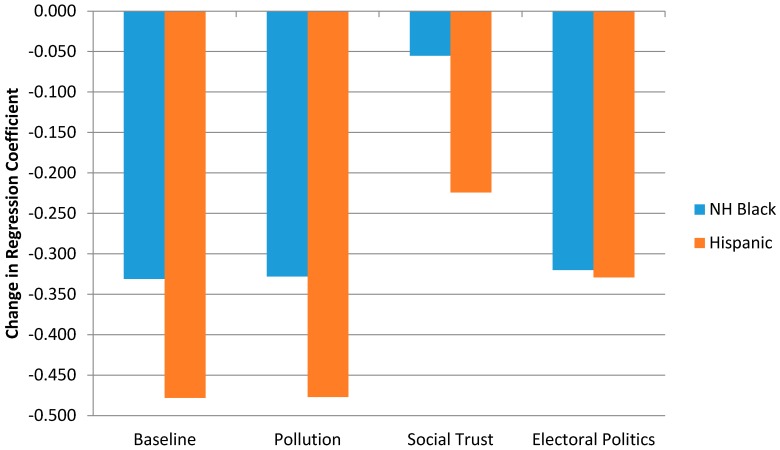
Changes in regression coefficient for an individuals’ race to predict self-rate health when accounting for differential pollution exposure and social capital.

As was previously noted when discussing findings from Table 3 (Models 4 and 8), general social trust explains the largest proportion of racial/ethnic disparities in self-rated health, reducing the Black/White gap by 84% and the Hispanic/White gap by 54%. Lower than average participation in electoral politics also seems to be an important mechanism through which health disparities by race/ethnicity are formed but more so for Hispanics than African Americans. Once we control for differential levels of this type of social capital, the size of the regression coefficient decreases by 32% (Figure 1).

When all three potential explanatory variables (pollution, social trust, and electoral politics) are simultaneously entered into regression analyses (Model 5), we find that the Black/White gap in self-rated health is reduced by such an order of magnitude that it ceases to be statistically significant (*p* < 0.05). This is largely driven by lower than average levels of social trust among African Americans. These findings suggest that certain forms of social capital, particularly those that are related to the trust an individual feels for neighbors of a different social standing and their perception of political empowerment, are critical characteristics of the social environment that drives racial/ethnic disparities in health, particularly among African Americans.

Regression results from Table 4 are also presented graphically in Figure 1 for ease of interpretation. These findings make it clear that, in these data, annual exposure to industrial air toxins does very little to help explain racial/ethnic disparities in self-rated health, while two of the seven social capital indicators we originally examined offer potential mediating mechanisms through which African Americans and Hispanics report significantly lower levels of self-rated health than Whites. Taking into consideration differential levels of participation in electoral politics across racial/ethnic groups reduces disparities in self-rated health for Hispanics to such a degree that their health status now resembles that of African Americans. The marked influence of social trust in the association between race/ethnicity and self-rated health is readily apparent. Once we adjust for significantly lower levels of social trust among African Americans and Hispanics, the Black/White gap in self-rated health all but disappears and the Hispanic/White gap is reduced by more than half.

## 4. Conclusions

This paper combines two unique national-level datasets on individuals nested within communities across the United States, and their estimated exposure to industrial air toxins, to estimate the extent to which two neighborhood mechanisms (social capital and annual estimates of exposure to industrial air toxins) can explain the racial gap in self-rated health. This paper is unique in that it is able to examine two possible mediators between race and health in a nationally representative sample while controlling for neighborhood context and individual health predictors. Across the U.S., non-White communities are at a greater risk of exposure to air pollution and social disadvantages. By examining these mechanisms in the same population, we are able to examine the relative effect of each in explaining racial disparities in self-rated health. 

Although African Americans had the highest estimated toxic exposure from industrial toxins, followed by Whites and Hispanics, controlling for relative exposure to air toxins was shown to have no impact on the racial gap in self-rated health. While we did not find support that annual estimates of residential exposure to industrial air pollution explained much of the racial gap in health, we did find support for the argument that social capital mediates the racial disparities in health. Firstly, we found that in general Hispanics were shown to have the lowest levels of social capital followed by African Americans and then Whites. While all measures of social capital were found to be significantly related to health outcomes, only two of these measures impacted the gap in self-rated health when controlling for individual covariates and community nesting. The strongest of these predictors, Cognitive Bridging, was reflected by an individuals’ general trust of those in their community who are not necessarily in their social groups (e.g., police, and shop employees). Once controlling for levels of Cognitive Bridging the gap in self-rated health between African Americans and Whites was no longer significant, decreasing the odds of an African American reporting they are in worse health than a White respondent by 84%. This supports the idea that some of African Americans’ greater propensity to report being in poorer health is linked to their feelings of trust, and social cohesion, of those in their community [28]. This also explained some of the gap between Whites and Hispanics, controlling for social trust decreased the odds Hispanics would report worse self-rated health by 54%. The other social capital indicator that was shown to explain some of the racial gap in health was also a cognitive dimension, Cognitive Linking. This was conceptualized as a feeling of political empowerment measured by activity and interest in politics. This measures decreased the odds Hispanics reported being in worse compared to Whites by 32% and its significance still held up in a full model (see Table 4, Model 5). Future work should follow-up this study by examining more direct measures of empowerment and stratifying by citizenship. 

Those measures that did not seem to explain the racial gap in health were: Informal Social Participation, Faith-Based Social Capital, Organizational Social Participation, Political Activism, Formal Group Involvement. We find it intriguing that the measures that made no impact on the racial gap in health were objectively measured, that is structural components of social capital, measured by the amount of time spent in activities, etc. While the two measures of social capital that did explain some of the racial gap in self-rated health were cognitive measures, thus measures that reflect feelings and values. We believe this suggests that psychology plays an important role in understanding how individuals’ social environment impacts their health. These findings hint that neighborhood characteristics might impact human health via psychological pathways, an argument reflected in the allostatic load theory [67], which poses that life’s daily stressors can accumulate in our body via biological pathways, such as secretion of cortisone. Future work should examine how the relationship between biomarkers for stress varies by perceived/cognitive and objective/structural measures of social capital as well as other objective health measures. Moreover, a thorough examination of how social capital interactions with other social and environmental factors should be undertaken. 

One surprising result from these analyses is that controlling for differential exposure to toxicity-weighted industrial air toxins had no impact on the gap in health between racial groups. One possible reason for this finding was raised by a recent review of the literature on social inequality and environmental inequality [68]. This review showed more dispersed air-pollutants, like those from industrial smoke stakes, were less strongly linked to health outcomes than more localized pollutants, like small industries and transportation, which were not estimated in these data [68]. Moreover, recent time use surveys show that 85% of working Americans work away from home which takes up an average of 7.8 h of their day [69]. This does not include commuting time, which exposes individuals to risk from a different set of air pollutants, largely exhaust or possible dangerous work conditions, which are more likely to be experiences by racial and ethnic minorities. Moreover, while our communities were comparable in population size to census block groups they did not follow census boundary lines but were instead determined by community members. In order to compare our results directly with much of the neighborhood effects literature future research should examine these mechanisms with neighborhoods redefined using census boundaries. Finally, the estimates of exposure to toxins were only for one year. The Weathering Hypothesis argues that for African Americans health begins to deteriorate at a faster rate than Whites due to compounding risk factors [70]. This would mean that, if we want to untangle the role that exposure to toxins has in this health gap, we might need cumulative exposures over time rather than a snapshot. To overcome these limitations, future work should incorporate different sources of air pollutants, as well as cumulative estimates and activity data to account for the multiple contexts in which people are exposed in their daily lives.

## Figures and Tables

**Table 1:** Measures of social capital.

Social Capital Index	Explanation	Social Capital Component				
		Bonding	Bridging	Linking	Cognitive	Structural
Informal Social Participation	A continuous index calculated as the mean of the responses to five questions, based on national survey norms: frequency of having friends visit, frequency of visiting with relatives, frequency of socializing with co-workers outside of work, frequency of hanging out with friends in public places, frequency of playing cards and board games. At least two of these questions had to be answered for a score to be calculated. The scores for each component part are standardized using U.S. parameters.	X				X
Faith-Based Social Capital	A sum of standardized measures of participation in organized religion: attendance, participate in church activities other than services, contributed money to church or religious causes, a church member, volunteered for religion, participated in organization affiliated with religion.	X				X
General Social Trust	An index comprising: general interpersonal trust, trust neighbors, trust co-workers, trust fellow congregants, trust store employees where you shop, trust local police. (At least three of these answers had to be provided for a score to be calculated.). Mean of the standardized responses to six questions, using national norms to standardize. Higher scores indicate higher social trust.		X		X	
Organizational Social Participation	A continuous index consisting of the factor score resulting from a principal components analysis of four components: number of formal group involvements (excluding church membership), serving as an officer or on a committee, number of club meetings attended, number of public meetings attended discussing school or town affairs.		X			X
Political Activism	A mean in seven different types of actions: belonging to any group that took local action for reform; attending a political meeting or rally in past 12 months; signing a petition in past 12 months; participating in political group; participating in demonstrations, boycotts, or marches in past 12 months; participating in ethnic, nationality, or civil rights organization; participating in labor union	X				X
Formal Group Involvement	A count of whether a respondent is affiliated with religion, sports club, league, or outdoor activity, youth organization, parent/school association, veterans group, neighborhood association, seniors groups, charity or social welfare organization, labor union, professional, trade, farm or business as, service or fraternal organization, ethnic, nationality, or civil rights org, political group, literary, art, or musical group, hobby, investment, or garden club, self-help program, a group that meets over the Internet.		X			X
Electoral Politics	This index reflects engagement with politics by combining the number of days in the past week the respondent read a daily newspaper, whether the respondent voted in the past election, is currently registered to vote, interested and knowledgeable of politics and national affairs.			X	X	

**Table 2:** Descriptive statistics on selected sociodemographic variables for all respondents and by race/ethnicity. Standard deviations are noted in parenthesis.

Variables	Total Sample	NH Whites	NH Blacks	Hispanics
*Self-Rated Health*				
Excellent	22.99	24.18	18.47	19.81
Very Good	36.91	38.85	33.46	26.03
Good	26.81	25.12	30.98	34.53
Fair	9.95	8.66	13.82	14.91
Poor	3.34	3.19	3.27	4.72
Pollution (decile)	5.56	5.48	6.53	4.77
	(2.85)	(2.81)	(2.68)	(3.03)
Informal Social Participation	−0.0067	0.0282	−0.0723	−0.1969
	(0.668)	(0.659)	(0.693)	(0.663)
Faith-based Social Capital	−0.0622	−0.0606	0.0821	−0.2885
	(0.748)	(0.761)	(0.717)	(0.620)
General Social Trust	0.0172	0.1735	−0.4862	−0.5277
	(0.695)	(0.603)	(0.708)	(0.751)
Organized Social Participation	−0.0009	0.0337	0.0483	−0.3587
	(0.993)	(0.989)	(1.069)	(0.825)
Political Activism	1.04	1.06	1.16	0.73
	(1.32)	(1.29)	(1.49)	(1.28)
Formal Group Involvement	2.96	3.01	3.40	1.96
	(2.65)	(2.51)	(3.18)	(2.59)
Electoral Politics	3.01	3.20	2.81	1.71
	(1.35)	(1.27)	(1.18)	(1.40)
Age	45.09	46.66	42.17	36.46
	(17.31)	(17.40)	(16.63)	(14.15)
*Race/Ethnicity*				
NH Whites	76.88			
NH Blacks	13.78			
Hispanics	9.34			
*Sex*				
Male	47.48	47.49	45.74	49.94
Female	52.52	52.51	54.26	50.06
*Region*				
Northeast	15.22	17.13	9.25	8.24
Midwest	31.48	33.59	31.35	14.30
South	30.18	28.50	45.82	20.98
West	23.12	20.78	13.58	56.48
*Marital Status*				
Never Married	23.26	20.08	34.95	32.21
Widowed	6.38	6.70	7.11	2.75
Divorced/Separated	12.87	11.91	18.68	12.23
Currently Married	57.49	61.32	39.27	52.81
*Education*				
Some High School	12.66	8.79	14.43	41.88
High School Graduate	28.11	27.86	30.48	26.72
Some College	23.19	23.36	27.87	14.89
Associate’s Degree	11.07	11.64	11.10	6.31
College Graduate	12.79	14.58	7.94	5.26
Graduate School	12.18	13.77	8.18	4.94
N	26,387	20,362	3512	2513

**Table 3:** Results from multilevel ordered logistic regression models predicting self-rated health.

	Model 1			Model 2			Model 3			Model 4			Model 5			Model 6			Model 7			Model 8		
NonHispanic White	Ref.			Ref.			Ref.			Ref.			Ref.			Ref.			Ref.			Ref.		
NonHispanic Black	−0.333	***	0.042	−0.311	***	0.043	−0.391	***	0.040	−0.055		0.042	−0.349	***	0.042	−0.338	***	0.043	−0.363	***	0.042	−0.322	***	0.043
Hispanic	−0.475	***	0.050	−0.428	***	0.047	−0.479	***	0.047	−0.218	***	0.045	−0.460	***	0.049	−0.471	***	0.049	−0.464	***	0.049	−0.322	***	0.045
Informal Social Interaction				0.147	***	0.028																		
Faith-Based Social Capital							0.264	***	0.019															
General Social Trust										0.530	***	0.028												
Organized Social Part.													0.117	***	0.013									
Political Activism																0.024	+	0.013						
Formal Grp Involvement																			0.043	***	0.006			
Electoral Politics																						0.180	***	0.011
N (Respondents)	26,387			26,387			26,387			26,387			26,387			26,387			26,387			26,387		

Source: Social Capital Benchmark Survey, 2000. Note: All models include additional control variables for age, sex, region, marital status, and education. Regression analyses were estimated using complex sampling weights. Robust standard errors were calculated using the Huber/White correction method and clustered at the community level. *** *p* < 0.001.

**Table 4:** Results from multilevel ordered logistic regression models predicting self-rated health.

	Model 1			Model 2			Model 3			Model 4			Model 5		
NonHispanic White	Ref.			Ref.			Ref.			Ref.			Ref.		
NonHispanic Black	−0.333	***	0.042	−0.330	***	0.042	−0.055		0.042	−0.322	***	0.043	−0.067		0.042
Hispanic	−0.475	***	0.050	−0.473	***	0.051	−0.218	***	0.045	−0.322	***	0.045	−0.118	**	0.042
Pollution				−0.006		0.004							−0.001		0.004
Social Trust							0.530	***	0.028				0.492	***	0.029
Electoral Politics										0.180	***	0.011	0.140	***	0.011
Age	−0.021	***	0.001	−0.021	***	0.001	−0.025	***	0.001	−0.027	***	0.001	−0.030	***	0.001
Sex	−0.001		0.024	−0.001		0.024	−0.029		0.024	0.027		0.024	−0.005		0.024
*Region*															
Northeast	Ref.			Ref.			Ref.			Ref.			Ref.		
Midwest	0.038		0.047	0.045		0.049	0.011		0.041	0.039		0.056	0.013		0.049
South	0.009		0.068	0.014		0.069	0.015		0.058	0.018		0.077	0.022		0.067
West	0.103		0.081	0.093		0.079	0.083		0.062	0.103		0.089	0.080		0.068
*Marital Status*															
Never Married	Ref.			Ref.			Ref.			Ref.			Ref.		
Widowed	−0.020		0.085	−0.021		0.086	−0.029		0.085	0.017		0.088	0.000		0.086
Divorced/Separated	−0.091	+	0.052	−0.092	+	0.052	−0.070		0.051	−0.066		0.052	−0.051		0.051
Currently Married	0.265	***	0.042	0.264	***	0.042	0.214	***	0.038	0.242	***	0.042	0.200	***	0.039
Education	0.281	***	0.009	0.281	***	0.009	0.243	***	0.009	0.231	***	0.009	0.207	***	0.009
N	26,387			26,387			26,387			26,387			26,387		

Note: Regression analyses were estimated using complex sampling weights. Robust standard errors were calculated using the Huber/White correction method and clustered at the community level. *** *p* < 0.001; ** *p* < 0.01; + *p* < 0.10.
